# Saccharomyces Cerevisiae Diet Supplementation Affects Nutrient Digestibility and Milk and Mozzarella Cheese Yield in Dairy Buffalo Cows During the Transition

**DOI:** 10.3390/ani14243689

**Published:** 2024-12-20

**Authors:** Fabio Zicarelli, Remus Costantin Vianello, Isabella Masiello, Nadia Musco, Piera Iommelli, Metha Wanapat, Daria Lotito, Pietro Lombardi, Micaela Grossi, Federico Infascelli, Raffaella Tudisco

**Affiliations:** 1 Department of Veterinary Medicine and Animal Production, University of Naples Federico II, 80137 Napoli, Italy; fabiozicarelli@gmail.com (F.Z.); vianelloremus@yahoo.it (R.C.V.); nadia.musco@unina.it (N.M.); daria.lotito@unina.it (D.L.); pilombar@unina.it (P.L.); micaelagrossi@tiscali.it (M.G.); infascel@unina.it (F.I.); tudisco@unina.it (R.T.); 2 Center for Applied Research in Agriculture (C.R.A.A.) Improsta, 84025 Eboli, Italy; isa.masiello1@gmail.com; 3 Tropical Feed Resources Research and Development Center (TROFREC), Department of Animal Science, Faculty of Agriculture, Khon Kaen University, Khon Kaen 40002, Thailand; metha@kku.ac.th

**Keywords:** Saccharomyces cerevisiae, buffalo cows, feed additive, transition period

## Abstract

The aim of the trial was to evaluate the influence of diet supplementation with Saccharomyces cerevisiae on nutrient digestibility and milk and mozzarella cheese yield in buffalo cows during the transition period. Twenty buffalo cows in the last month of pregnancy were equally divided into two groups (control, C, and treated, T) homogeneous for parity and milk yield at the previous lactation. The diet of group T was supplemented with 100 g/head/day of a product containing Saccharomyces cerevisiae (Sc 47- CNCM I-4407) for 4 weeks before and after calving. Dry matter intake and the body condition score were not affected by the dietary treatment. In contrast, organic matter, protein, NDF, and ADF digestibility significantly (*p* < 0.01) increased. Group T showed a higher milk yield (kg 10.5 vs. 9.2, *p* < 0.05) but lower milk fat (g/kg 76.0 vs. 80.1, *p* < 0.05). The buffalo standard milk (FPCM = 740 kcal) yield was higher in the treated group (kg 16.8 vs. 15.0, *p* < 0.05), whereas the mozzarella cheese yield was not affected by the treatment (*p* > 0.05). These findings indicate that supplementation of the diet of buffaloes with Saccharomyces cerevisiae during the transition period results in notable enhancements in nutrient digestibility and milk yield. These outcomes are worthy of further investigation.

## Simple Summary

Due, in large part, to the popularity of mozzarella cheese, buffalo breeding has become quite popular in Italy in the new century. The transition (30 days before to 30 days after calving) is a critical period for buffalo cows, with a decrease in dry matter intake and a consequent loss of body weight. These phenomena lead to a higher susceptibility of buffalo cows to metabolic disorders, which may compromise their performance. Following the prohibition of antibiotics for auxinic purposes, there has been a surge in interest in feed additives and their potential to improve animal performance and nutrient use through effects on rumen metabolism. Saccharomyces cerevisiae (yeast) cultures have garnered significant attention as supplements in ruminant nutrition. Therefore, the influence of diet supplementation with Saccharomyces cerevisiae on nutrient digestibility and milk and mozzarella cheese yield in buffalo cows during the transition period was investigated.

## 1. Introduction

In Italy, buffalo (Bubalus bubalis) breeding has high economic importance, mainly linked to the production of mozzarella cheese [1,2] and, to a lesser extent, to buffalo meat, because of the growing interest of consumers in recent years [3]. Compared to bovines, buffaloes show several peculiar traits for digestive physiology [4], which allows a better use of fibrous feeds [5]. Nevertheless, aiming to increase the milk yield (MY), buffalo breeders are adopting intensive diets that negatively influence rumen fermentation [6].

Similar to dairy cows, the transition (30 days before to 30 days after calving) is a critical period for buffalo cows also, with a decrease in dry matter intake (DMI) and a consequent loss of body weight [7]. Therefore, the improvement of feed use during the transition period is a goal to avoid the above-mentioned disorders. In this context, the supplementation of the diet with modulators of rumen fermentation may be useful. According to Zhang et al. [8], probiotics have a favorable effect on animal health. Among them, yeast (Saccharomyces cerevisiae, SC) can be supplemented in ruminant rations to promote rumen fermentation [9,10]; indeed, SC furnishes growth factors to cellulolytic bacteria, enhancing fiber degradation [11]. Several authors have reported an improvement in the performance of lactating dairy cows due to the supplementation of diets with SC [12,13], even if other authors have found contrasting results [14,15]. These differences could be due to the diet characteristics or to the productive phase of the animals, with a more positive effect at the beginning of lactation [16]. According to Backr et al. [17], in yeast-supplemented dairy cows, the MY and fat percentage are higher.

Compared to cows, research on buffaloes is poor and often not exhaustive. Management improvements in buffaloes, including diet supplementation, are often based on studies performed on bovines, despite the above-mentioned differences between the two species. In early-lactating dairy buffaloes, Infascelli et al. [18] supplemented the diet with SC, obtaining an increase in the MY, and attributed such results to higher fiber fermentation with an increase in volatile fatty acid (VFA) production and a lower availability of H2 for methane production, thus lowering energy losses [17]. Moreover, Campanile et al. [19] reported an improvement in organic matter digestibility mainly during the first phase of lactation (<135 days), with an increase in the body condition score and MY. These results were also confirmed by other authors [20,21], who reported a significant increase in diet digestibility, while fecal nitrogen excretion significantly decreased [20] and the DMI and MY and fat-corrected milk yield increased [21] in early-lactating buffaloes fed a diet supplemented with SC. According to Hansen et al. [22], the response to SC supplementation in buffaloes is parity dependent; an increase in the DMI and fiber digestibility was observed in multiparous cows, as well as an improvement in the MY during early lactation. In addition, Di Francia et al. [23], supplementing the diet of buffalo calves with Aspergillus oryzae fermentation extract and SC, observed an improvement in digestive efficiency.

As seen, research on buffaloes is quite dated, without reaching a definitive point on the usefulness of SC supplementation in buffaloes during the transition period, which represents, also in buffaloes, a critical period that has not been fully investigated in previous research. For these reasons, the aim of the present trial was to evaluate in dairy buffalo cows the effects of diet supplementation with SC, a yeast probiotic, during the critical transition period on the DMI, diet digestibility, and milk and mozzarella cheese yield. The hypothesis was that such supplementation could positively affect feed use, ameliorating the animals’ milk yield and quality during early lactation.

## 2. Materials and Methods

All procedures involving animals were approved by the Ethical Animal Care and Use Committee of the University of Napoli Federico II (Prot. 2019/0013729 of 8 February 2019).

The trial was performed in February and March 2023 at the Experimental Farm “Improsta” of the Campania Region (Eboli, Salerno province, 145 m a.s.l. 40°37′1#x2033; N, 15°3′23″ E, with 842 mm average rainfall and 11.3–19.5 °C mean temperature).

### 2.1. Animals and Diets

Twenty buffalo cows in the last month of pregnancy were equally divided into two groups (C, control, and T, treated) homogeneous for parity (3 to 5 calving), body weight (BW, kg 632 ± 23), and previous MY (kg 2290 ± 132). They were allocated to individual open yards with a permanent bedding area (7 m^2^/head), an exercise area (10 m^2^/head), and a feeding area (3 m^2^/head) and with free access to water. All animals were individually fed a diet for dry buffalo cows, composed of 7.5 kg of wheat straw, 2.8 kg of wheat middlings, 150 g of salts (calcium–phosphorus 1:3), and 40 g of a vitamin complex (ADE). To the diet of group T was added 100 g/head/day of a product (Transition SAF, VITASOL, Brescia, Italy) constituted of wheat gluten feed, barley meal, CaCO3, and a vitamin–mineral complex and containing SC (Sc 47-CNCM I-4407 1000 mld CFU). The yeast supplement was top-dressed onto the morning feed.

**Figure 1:**
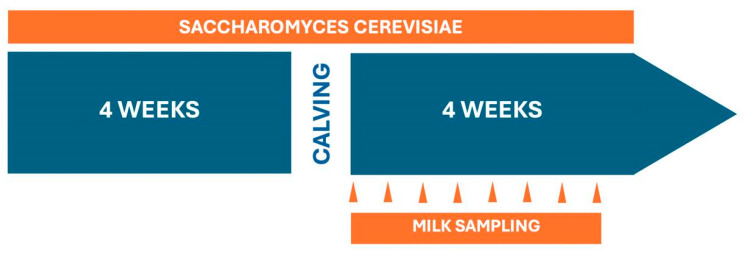
Timeline of experimental activities.

After calving, all buffalo cows received 16 kg/head/day, as the total mixed ration (TMR), of the diet reported in Table 1, which for group T was supplemented (100 g/head/day) with the above-mentioned product containing SC. In Figure 1, the timeline of the experimental activities is presented.

The buffalo cows’ body condition score (BCS) was evaluated weekly on a 1-to-5 scale, where 1 = emaciated, 2 = thin, 3 = average, 4 = fat, and 5 = obese, as reported by Anitha et al. [24].

### 2.2. Diet Analysis and Digestibility

From the feed fence of each animal, samples of the TMR were collected weekly before the animals’ feeding and analyzed for the chemical composition according to the AOAC [25]. Briefly, samples milled to pass through a grid of 1.1 mm were analyzed for dry matter (DM), crude protein (CP), and ether extract (EE) contents (ID number: 2001.12, 978.04, 920.39 and 978.10, 930.05, respectively), while neutral detergent fiber (NDF), acid detergent fiber (ADF), and acid detergent lignin (ADL) were analyzed as suggested by Van Soest et al. [26].

The starch content was determined by the polarimetric method (Polax L, Atago, Tokyo, Japan) as per the official procedure (ISO 6493:2000 28). The diet energy value (forage unit for lactation (UFL): 1700 kcal) was calculated according to the INRA [27].

In addition, TMR granulometry was measured by using three meshes (the first retaining particles above 19 mm holes; the 2nd, those above 8 mm; and the 3rd, particles above 1.18 mm) and the peNDF calculated by the Penn State University method [28].

TMR refusals were collected daily to calculate the individual DMI.

During the last 5 days of the trial, fecal samples (200 g) were collected from the rectum of each buffalo cow four times a day and analyzed, as reported before for the diets. Organic matter (OM), EE, CP, NDF, and ADF in vivo digestibility was determined by using acid-insoluble ash (AIA) as the internal marker [29].

### 2.3. Milk

Immediately after calving, the individual MY was measured daily (by TDM software version 4.1), while individual milk samples were collected two times a week for 4 weeks and analyzed for the chemical composition, urea, and caseins (Milko Scan 133B, Foss Matic, Hillerod, Denmark). Buffalo standard milk (fat- and protein-corrected milk (FPCM) = 740 kcal) was calculated by the following equation [30]:

([{fat (g/kg) − 40 + protein (g/kg) − 31}0.01155] + 1) MY.

Finally, the mozzarella cheese yield (MCY) was calculated by the formula of Altiero et al. [31]:

MCY (kg) = [3.5 (% protein) +1.23 (% fat) − 0.88]/100

### 2.4. Statistical Analysis

Data were analyzed by using the two-way ANOVA procedure of JMP^®^ (version 14; SAS Institute, Cary, NC, USA) according to the following model:

yijk = m + Gi + Sj +GxSij + d(Gi) + eijk

where yijk = single observation, m = general mean, Gi = group effect (i = C and T), Sj = sampling effect (j = I, II, III……VIII), GxS = interaction between group effect and sampling effect, d(Gi) = random effect of a buffalo within a group, and eijk = experimental error.

A comparison among the mean values was performed by using Tukey’s test.

The differences were considered significant at *p* < 0.05. All the statistical procedures were performed using JMP software (version 14; SAS Institute, Cary, NC, USA).

## 3. Results and Discussion

The chemical composition of the TMR is reported in Table 2. The energy values and protein concentration of the diet were adequate for lactating buffalo cows [32]. Finally, the PeNDF content, in particular its incidence on the total NDF, was appropriate for buffalo cows in the first phase of lactation [33].

No feed refusals were detected. The DMI was not different between the groups (Table 3).

Variable values of the DMI for buffalo cows have been reported, ranging from 2.2–2.6% [34] to 2.7 and 3.4% body weight [30]. According to Campanile et al. [19], the DMI/kg FPCM is equal to 275 g, in addition to the amount required for maintenance (91 g/kg body weight 0.75) when diets show NDF/DM between 45 and 49%. The DMI is lower in the first 50 days of lactation, leading to live weight loss, as in dairy cows, even if the lower catabolic activity of buffaloes results in a decrease in the MY, if nutritive requirements are not satisfied [32]. In buffaloes, the DMI is more homogeneous than in bovines during the day, mainly with a high-fibrous diet, probably due to the more extended mastication [35,36]. Furthermore, buffaloes eat faster than cattle due to their more developed incisive teeth [19]. Also, they show a faster digestive passage rate [37] and higher organic matter use by rumen microorganisms [38,39,40,41,42]. In our trial, the lack of differences in the DMI between groups certifies the good palatability of the experimental products; in fact, the buffalo species is known to be wary of new feeds in the diet [32]. Contrasting results have been reported by Sinclair et al. [43] in dairy cows who found an increased DMI and suggested multiple alterations in the rumen due to SC supplementation, including a rise in pH, modifications in volatile fatty acid proportions, and improved catabolism of fibrous carbohydrates. Nevertheless, those authors tested a different strain of SC and administered a different dosages to the animals.

The organic matter, crude protein, starch, NDF, and ADF digestibility was significantly (*p* < 0.01) increased by SC supplementation of the diet (Table 3), as also reported by others [23,44]. These results should be attributed to the improved rumen ecosystem either by the production of digestive enzymes [45] or by the stimulation of proteolytic bacteria, thus increasing crude protein catabolism [11]. Despite the improved nutrient digestibility, the SC supplementation did not affect the BCS, which was similar between the groups according to Dann et al. [46] and Ali et al. [47].

**Figure 2:**
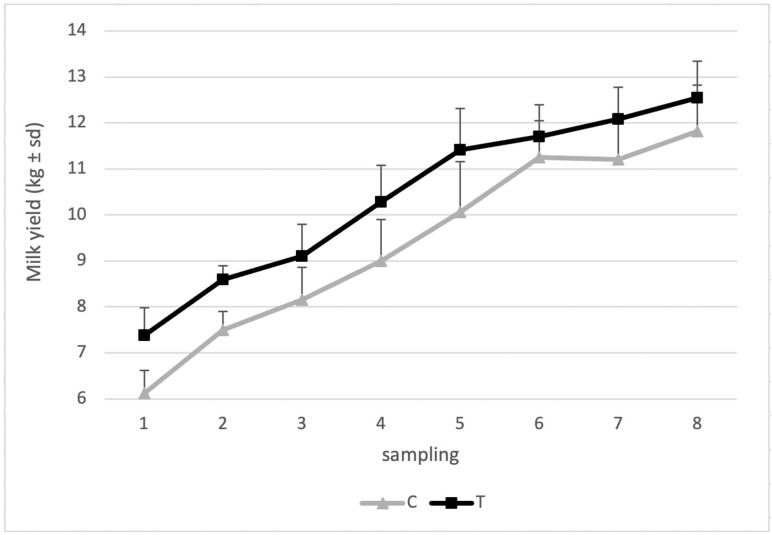
Milk yield of the control (C) and treated (T) groups during the trial. Milk samples were collected two times a week for 4 weeks.

Diet supplementation with products containing SC significantly increased the daily MY (kg 10.5 vs. 9.2 for groups T and C, respectively; *p* < 0.05) (Table 4) and significantly decreased milk fat (g/kg 76.0 vs. 80.1 for groups T and C, respectively; *p* < 0.05), whereas no significant differences were observed for the milk protein and lactose content. Nevertheless, supplementation resulted in a significant increase in the FPCM (kg 16.8 vs. 15.0 for groups T and C, respectively). Milk urea and casein, as well as the mozzarella cheese yield, were not significantly affected by the diet supplementation. As shown in Figure 2, grouping the data of 5 consecutive days, the MY of the treated group was higher than that of the control group during the entire trial.

Several authors have reported an increase in the MY by supplementing ruminants’ diets with SC [48,49]. In contrast, Szucs et al. [50] and Zicarelli et al. [51] did not observe any influence on MY due to diet supplementation with probiotics. In buffaloes, SC supplementation of the diet significantly improved the MY (up to 6.7%) and milk fat and protein [23,44]. The contrasting results could be attributed to the animal species and to the different trial conditions, particularly the physiological stage, the productive level, the feeding system, and the energy and protein concentration of the diets. In this regard, Miranda et al. [52] compared diets with two levels of NDF (27 vs. 37%) and observed a significant (*p* < 0.05) influence of the probiotics only for the diet with 37% NDF. According to Yuan et al. [53], SC supplementation increases energy efficiency, thus reducing maintenance requirements and increasing the use of net energy for milk production.

**Figure 3:**
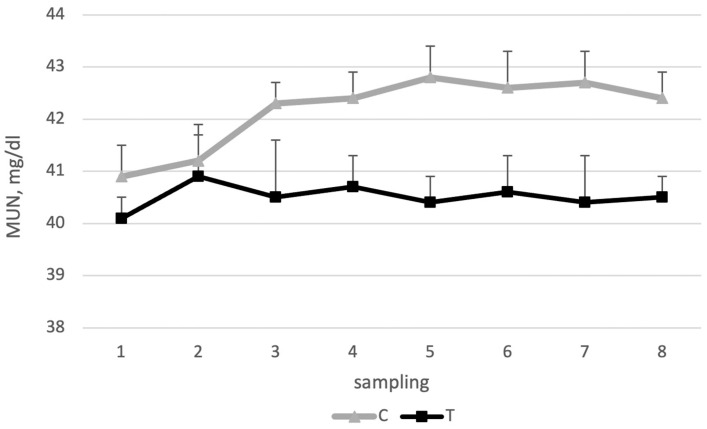
MUN of the control (C) and treated (T) groups during the trial. Milk samples were collected two times a week for 4 weeks.

As depicted in Figure 3, milk urea showed lower levels in group T (40.5 vs. 42.2% for groups T and C, respectively). Although the difference was not significant (*p* = 0.087), such a tendency may reflect improved nitrogen use due to the modulating activity of the probiotic on rumen fermentation [54], rather than a different efficiency in protein digestion.

Indeed, in this trial, the diet was supplemented for a relatively short time, and a further influence of SC when used for longer duration should not be excluded. In any event, our results confirm the higher milk urea concentration in buffaloes than in dairy cows due to the greater efficiency of the urea cycle mechanism in buffaloes [55].

## 4. Conclusions

In this research, supplementation of the diet of buffalo cows with 100 g/head/day of a product containing SC in the transition period resulted in a significant improvement in nutrient digestibility and milk yield but a lower percentage of milk fat. In addition, the dry matter intake, body condition score, and MCY were not affected, thus confirming the importance of diet digestibility in improving production in buffaloes and suggesting that SC may be used to obtain better feed use. Further investigation may better define important features, such as the optimal amount and time of supplementation, to achieve the best results.

## Figures and Tables

**Table 1:** Ingredients (kg/DM) of the TMR for lactating buffalo cows.

Corn silage	3.8
Alfalfa haylage (1st cut)	3.0
Corn mash	2.7
Polyphite hay *	1.9
Concentrate **	4.1
Hydrogenated fats plus vitamins	0.2
Salts	0.06

* *Phleum pratense* L., *Lolium italicum* L., and *Trifolium pratense* L. ** Wheat middling, soybean meal, corn meal, carob meal, barleymeal, soft wheat meal, and sunflower meal.

**Table 2:** Chemical composition (g/kg DM) of the total mixed ration during the lactation period.

(g/kg DM)	Diet
Crude protein	154.2 ± 15.3
Ether extract	44.0 ± 10.4
NDF	383.6 ± 24.3
ADF	209.2 ± 16.2
ADL	57.2 ± 9.1
PeNDF	276.1 ± 16.2
Starch	223.2 ± 14.1
Ash	72.4 ± 11.2
UFL (kg DM)	0.9

**Table 3:** Body condition score (BCS), dry matter intake (DMI, kg/day), and nutrient digestibility (%) of the two groups during the lactation period.

Item	Control	Treated	*p*-Value
BCS	3.2 ± 0.3	3.3 ± 0.2	NS
DMI (kg/day)	14.5 ± 1.5	14.5 ± 1.8	NS
*Digestibility (%)*			
Organic matter	58.2 ± 3.1	68.1 ± 2.2	<0.01
Crude protein	55.3 ± 1.8	63.9 ± 1.7	<0.01
Ether extract	88.2 ± 1.1	88.8 ± 1.6	NS
NDF	37.9 ± 2.7	49.6 ± 2.1	<0.01
ADF	34.3 ± 3.1	42.3 ± 2.9	<0.01
Starch	89.3 ± 1.2	96.8 ± 1.1	<0.01

NS: not significant.

**Table 4:** Milk yield (MY) (kg ± sd), milk chemical composition (g/kg ± sd), FPCM yield (kg ± sd), milk urea (MUN, mg/dl ± sd), caseins (% ± sd), and mozzarella cheese yield (MCY, % ± sd) of the control (C) and treated (T) groups.

Item	Control	Treated	*p*-Value
MY	9.2 ± 2.8	10.5 ± 1.9	<0.05
Fat	80.1 ± 1.3	76.0 ± 1.0	<0.05
Protein	46.1 ± 0.3	47.1 ± 1.5	NS
Lactose	45.0 ± 0.2	44.8 ± 1.4	NS
FPCM	15.0 ± 3.1	16.8 ± 3.6	<0.05
MUN	42.2 ± 0.9	40.5 ± 0.7	NS
Caseins	4.0 ± 0.3	4.1 ± 1.3	NS
MCY	25.2 ± 2.6	24.8 ± 7.6	NS

NS: not significant.

## Data Availability

Data are available from the corresponding author upon reasonable request.
